# A Randomized Controlled Trial Comparing Soy-Pea Protein to Animal Protein in Adults with Crohn’s Disease

**DOI:** 10.64898/2026.05.20.26353678

**Published:** 2026-05-20

**Authors:** Abigail Raffner Basson, Jeffry Katz, Vu Nguyen, Drishtant Singh, Paola Menghini, Adrian Gomez-Nguyen, Jennifer Sieg, Madison Bell, Kavitha Thamma, Gina Ponzani, Abdullah Osme, Alexander Rodriguez-Palacios, Fabio Cominelli

## Abstract

**Background and Aims:**

Diet plays a critical role in managing Crohn’s disease (CD) inflammation. We assessed whether dietary replacement of animal protein (AnimalP) by soy-pea protein (SoyP) decreases the pro-inflammatory potential of gut microbiota and intestinal inflammation in CD patients.

**Design:**

In an open-label, randomized controlled feeding trial at University Hospitals Cleveland Medical Center, CD participants and healthy controls were randomized (1:1) to a soy-pea or animal protein diet for 7-days. Primary outcomes were the absolute difference (Δd7-d0) in; Crohn’s Disease Activity Index (CDAI) score and fecal myeloperoxidase (MPO). Secondary outcomes included fecal calprotectin (FC) and high-sensitivity C-reactive protein (hsCRP). Murine fecal transplantation experiments were performed to determine the inflammatory potential of diet-altered gut microbiota.

**Results:**

The study randomized 66 participants and 60 were included in the final analysis (n=31 CD, n=29 HC). After 7 days, CD-SoyP participants were more likely than CD-AnimalP to show reductions in HBI (RR=4.68, 95% CI: 1.22-17.98, P=0.009) and fecal MPO (RR=2.30, 95% CI: 1.04-4.85, P=0.032), with a similar directional trend for CDAI (RR=1.52, 95% CI: 0.89-2.58, P=0.135). No participants experienced worsening of CDAI. The rank-based composite CDAI-MPO score was lower in the CD-SoyP vs CD-AnimalP group (median [IQR]: 5 [4-6] vs 8 [7-9]; P=0.012). Stratified analyses showed significant reductions in fecal MPO among CD participants with lower baseline disease activity (CDAI <150; P<0.0001), but not in those with higher activity (P=0.799)

**Conclusion:**

Short-term addition of plant-based soy-pea protein within a controlled diet exerted a beneficial, anti-inflammatory effect in CD, with evidence of greater effects among participants with lower baseline disease activity. ClinicalTrials.gov, Number NCT04065048 .

## Full Text Availability

The license terms selected by the author(s) for this preprint version do not permit archiving in PMC. The full text is available from the preprint server.

