# Supplementary material for: A Randomized Controlled Trial Comparing Soy-Pea Protein to Animal Protein in Adults with Crohn’s Disease
Source: medRxiv. 2026 Jun 11:2026.05.20.26353678. Originally published 2026 May 20. Preprint. [Version 2] doi: 10.64898/2026.05.20.26353678 (PMC13228749; doi:10.64898/2026.05.20.26353678)
Supplement: Supplement 1 [file media-1.pdf]

Supplemental Files

A Randomized Controlled Trial Comparing Soy-Pea Protein to Animal Protein in Adults with Crohn's Disease

Abigail Raffner Basson[1,2,3*], Jeffry Katz[4], Vu Nguyen[4], Drishtant Singh[1,2], Paola Menghini[1,2], Adrian Gomez-Nguyen[1,2], Jennifer Sieg[1], Madison Bell[1,2], Kavitha Kamma[1,2], Gina Ponzani[1,2], Abdullah Osme[1,5], Alexander Rodriguez-Palacios[1,2], Fabio Cominelli[1,2]

[1] Division of Gastroenterology & Liver Diseases, Case Western Reserve University School of Medicine, Cleveland, OH, USA.
[2] Digestive Health Research Institute, University Hospitals Cleveland Medical Center, Cleveland, OH, USA.
[3] Department of Nutrition, Case Western Reserve University School of Medicine, Cleveland, OH, USA.
[4] Digestive Health Institute, University Hospitals Cleveland Medical Center, Cleveland, OH, USA.
[5] Department of Pathology, University Hospitals Cleveland Medical Center, Case Western Reserve University School of Medicine, Cleveland, OH, USA

Correspondence:
Abigail Raffner Basson (axb860@case.edu, 0829ab@gmail.com)
2109 Adelbert Road, Biomedical Research Building 9th floor, Cleveland, OH, 44106
Tel: +1(216) 630-0365

Table of Contents

A. Supplementary Methods

Assessment of Participants
Patient and Public Involvement
Adherence and Macronutrient Intake
Adverse Events
Protocol Deviations
Protocol Modifications
Monitoring and Data Quality
Laboratory Analysis
Fecal Microbiota Transplantation Experiments in Mice

B. Supplementary Discussion

C. Supplementary Tables

Supplementary Table 1. Self-reported Foods Consumed by Participants on the Soy-pea Protein Diet that were Not Provided by the Study
Supplementary Table 2. Self-reported Foods Consumed by Participants on the Animal Protein Diet that were Not Provided by the Study
Supplementary Table 3. Within-group Total Daily Macronutrient and Fiber Intake Among Males
Supplementary Table 4. Within-group Total Daily Macronutrient and Fiber Intake Among Females
Supplementary Table 5. Between-group Total Daily Macronutrient and Fiber Intake Among Males
Supplementary Table 6. Between-group Total Daily Macronutrient and Fiber Intake Among Females
Supplementary Table 7. Within-group Total Micronutrient Intake Over 7 Days Among Male Crohn's Disease Participants by Diet (SoyP vs AnimalP)
Supplementary Table 8. Within-group Total Micronutrient Intake Over 7 Days Among Male Healthy Control Participants by Diet (SoyP vs AnimalP)
Supplementary Table 9. Within-group Total Micronutrient Intake Over 7 Days Among Female Crohn's Disease Participants by Diet (SoyP vs AnimalP)
Supplementary Table 10. Within-group Total Micronutrient Intake Over 7 Days Among Female Healthy Control Participants by Diet (SoyP vs AnimalP)
Supplementary Table 11. Between-group Total Micronutrient Intake Over 7 Days Among Male Crohn's Disease and Healthy Control Participants Assigned the SoyP diet
Supplementary Table 12. Between-group Total Micronutrient Intake Over 7 Days Among Male Crohn's Disease and Healthy Control Participants Assigned the AnimalP diet
Supplementary Table 13. Between-group Total Micronutrient Intake Over 7 Days Among Female Crohn's Disease and Healthy Control Participants Assigned the SoyP diet
Supplementary Table 14. Between-group Total Micronutrient Intake Over 7 Days Among Female Crohn's Disease and Healthy Control Participants Assigned the AnimalP diet

D. Supplementary Figure

Supplementary Figure 1. Fecal microbiota transplantation experiments using baseline ("pre") and diet-altered ("post") stool collected from Crohn's disease participants assigned to the SoyP-diet intervention.

E. Supplementary References

A. Supplementary Methods

Assessment of Participants

We used a combination of participant self-reported outcomes and medical records for demographic information, CD history, medical and medication history, dietary recalls, and disease symptoms. Disease activity was measured using the Crohn's Disease Activity Index (CDAI, primary outcome) and HBI. Fecal MPO, fecal calprotectin (FC), and high-sensitivity C-reactive protein (hsCRP) were measured to assess the inflammatory response to the diet. Fecal calprotectin (FC) analysis was conducted in a single batch at the conclusion of the study. Final analyses of FC data excluded one CD AnimalP-diet participant due to an erroneous value quantified by the kit.

Our group has demonstrated in animal models and humans that fecal MPO changes in response to diet[1], and represents an optimal marker to assess the inflammatory response to diet. In the present study, we used fecal MPO as a surrogate marker to evaluate changes in intestinal inflammation in CD patients following the dietary intervention. We and others have shown that the presence of neutrophils in feces, quantified by MPO activity (a primary constituent of neutrophils) and by fecal calprotectin (FC), serve as a reliable indicator for monitoring intestinal inflammation in IBD.

Patient and Public Involvement

Patients and the public were not involved in the development of the research question, outcome measures, or study design. The research questions and endpoints were informed by prior preclinical and translational work. Participants were first involved at the point of recruitment and participation in the study. Although patients were not involved in the design or conduct of the study, efforts were made to minimize participant burden. At the conclusion of the study, participants completed a diet satisfaction survey to assess acceptability and perceived burden of the intervention. Study findings will be disseminated to participants through a lay-language summary of the results, and efforts will be made to share findings with relevant patient communities in accessible formats. Future studies will incorporate patient and public involvement in the design and refinement of research questions, outcome measures, and dissemination strategies to better align with patient priorities and preferences.

Adherence and Macronutrient Intake

Foods that were consumed but which were not provided by the study diet were determined as either 'compliant' or 'non-compliant' based on the food type. For example, the consumption of cow's milk (if not provided by the study) by a participant on the AnimalP-diet would be deemed 'compliant', but deemed as 'non-compliant' for a participant on the SoyP-diet. Overall, only one participant on the SoyP-diet reported a single occurrence of consuming a non-compliant food item (skim milk). Foods consumed that were not provided by the study are shown in **Supplementary Tables 1-2.**

Adverse Events

Adverse events were assessed at end of study visit (day 7 of the study diet). Serious adverse events included any adverse event that was fatal, life-threatening, requiring prolonged hospital stay, congenital anomaly or birth defect or other medically significant event as deemed such by the investigator.

Overall, most of the adverse events reported by participants at the end of study visit were gastrointestinal related, and resolved within the first 1-2 days of the study diet. Bloating/flatulence was the most common complaint reported (SoyP diet (CD; 12.5%, 2/16, HC; 28.5%, 4/14) vs AnimalP (CD; 20%, 3/15], followed by constipation (SoyP; CD 6.25%, 1/16) and increased stool (SoyP; CD 6.25%, 1/16). Headache was reported by one CD SoyP and one CD AnimalP participant.

A total of two adverse events that were possibly related to the study diet were reported (attrition rate of 6.6%, n=2/30 participants in the SoyP-diet groups; 1 CD and 1 healthy control participant). In brief, one female Crohn's disease (CD) participant assigned to the soy-based diet experienced abdominal discomfort and nausea in the evening of day 1 after starting the soy-based diet. The participant was instructed to immediately stop the study diet and symptoms resolved within 48 hours. The second additional adverse event was reported in one female HC participant assigned to the soy-based diet who reported experiencing a headache and nausea after starting the study diet. However, the participant reported "I believe I experienced a headache, low levels of energy and nausea as a result of not consuming enough calories during the day and not receiving my normal protein intake". No other adverse events were reported. No Deaths or other Unanticipated problems were reported.

Protocol Deviations

Protocol deviations that occurred early in the study included: (1) Improper completion of the nurse flow sheet during visit 1 for one participant, (2) use of an outdated version of the consent form during the consenting process for six participants, and (3) initiation of the study diet more than 14 days after stool sample 1 was collected for one participant. In addition, although the protocol specified an HBI score <4 or >8, CD participants with scores between 5-7 were included. None of these deviations impacted the completeness, accuracy, or reliability of the study data.

Protocol Modifications

This study began enrolling in September 2019, just before the onset of the COVID-19 pandemic. Due to health mandates implemented during the pandemic, study activities were temporarily halted. To ensure the continuation of the study while minimizing the burden of in-person hospital visits, the following amendments were made to the study protocol: (1) Consent and study procedures, initially planned as in-person, were conducted remotely via Doxy.me, following the "Guidelines for Remote Electronic Consent" provided by University Hospitals Cleveland Medical Center, and (2) Blood draws were arranged to take place at participants' homes through the local home health care company BrightStar Care.

The original study was developed based on a 2019 Cleveland Digestive Diseases Research Core Center (DDRCC) pilot feasibility award (P30DK097948) and initially focused exclusively on individuals with Crohn's disease (CD). However, in 2020, the principal investigator (ARB) secured additional funding (K01DK127008-01), which enabled the expansion of the study to include healthy controls (HC) and an increase in the proposed sample size from 12 CD participants to 60 subjects (30 CD and 30 HC). Additionally, fecal myeloperoxidase (MPO) activity was incorporated as a primary outcome to allow comparisons between Crohn's patients and controls. The quantification of fecal MPO had already been approved in the original protocol. Initially, the study's inclusion criteria required participants to be between 18 and 55 years old. After five months of recruitment, the study steering committee expanded the age range to 18-65 years to broaden eligibility. Furthermore, an additional gastroenterologist was added to the study as an investigator to support expanded patient recruitment efforts.

Monitoring and Data Quality

This study was overseen by a Data Safety Monitoring Board (DSMB), which convened at three key points: prior to the study's initiation, after half of the participants were enrolled, and at the conclusion of the trial. The monitoring focused on regulatory documentation, the consent process, participant eligibility and interim analyses of data. DSMB meetings followed a standard format, starting with an open session involving both DSMB members and study investigators. This was followed by a closed session where unblinded data could be reviewed, and, if necessary, an additional open session was held. In December 2022, the study underwent a comprehensive research compliance audit conducted by University Hospitals. The audit found the study to be in good standing, with only minor compliance issues noted. All required actions from the audit's final report were addressed, resolved, and documented in the study's regulatory binder.

Laboratory Analysis

Blood serum was collected and analyzed for hematocrit and high-sensitivity C-reactive protein (hsCRP) by University Hospitals Laboratory Services. Freshly voided stool samples were stored at -80°C within 2 hours of collection. Fecal MPO activity was assessed (measured in triplicate) within 5 days of collection, as previously described[2, 3].

Fecal Microbiota Transplantation Experiments in Mice

To understand the role of microbiome changes on inflammation, we conducted a series of fecal microbiota experiments. Groups of six 14-week-old sex-matched (littermate controls) inbred germ-free (GF) SAMP1/YitFC (SAMP) mice (Cleveland Digestive Diseases Research Center, Mouse Models Core) transplanted with either 'pre' (d0) or 'post' (d7) SoyP or AnimalP diet-altered human feces were used to quantify the inflammatory (functional) potential diet-altered human gut microbiota. Details on the human gut microbiota transplanted GF SAMP (hGM-SAMP) model is previously described[4]. Two weeks post colonization, colitis was induced with 3% DSS (TdB Consultancy AB) offered ad libitum for 7 days and mice resumed with water for 2 days, then were killed. The impact of FMT was assessed using five parameters associated with colonic inflammation (MPO, FITC, colon length, and histology).

We have demonstrated that germ-free (GF) SAMP mice transplanted with human gut microbiota (hGM-SAMP) and treated with dextran sodium sulfate; DSS (3% for 7days) display increased susceptibility to DSS-associated morbidity depending on the 'pro-inflammatory' potential of human donor feces[4, 5]. Concurrent reports

support temporal differences in the IBD microbiome[6]. We used our validated hGM-SAMP DSS model as a model of gut barrier dysfunction (described in detail[4, 5]) to quantify the inflammatory (functional) potential of SoyP and AnimalP diet-altered human gut microbiota. Fecal myeloperoxidase (MPO) activity was assessed in mouse feces (measured in triplicate) as described[3, 7]. Intestinal permeability was measured using a fluorescently labeled small molecule [fluorescein isothiocyanate (FITC)-dextran], as described[8]. Colonic tissue samples were submitted for blinded histological assessment of formalin-fixed tissues using validated methodology[2, 9]. Murine colonoscopy was performed on the day of sacrifice using a flexible digital ureteroscope (URF-V; Olympus America) and inflammation quantified using a validated endoscopic scoring system[10].

Animals were maintained on a standard rodent chow following weaning. Importantly, animals were not fed soy- or animal-protein diets resembling the human interventions, in order to determine whether the hypothesized microbiome-mediated anti-inflammatory effects observed in humans could also be detected in mice when maintained on a different diet. Thus, throughout the experiment, all mice were fed laboratory rodent pellets, standard to our facility (Labdiet® Rat/Mouse 18%VacPac-5LQ6, Charles River), and which have been used in our facility to characterize the SAMP mouse CD-like ileitis phenotype in various studies[2, 7, 11]. All diets were vacuum packed and double irradiated to reach GF standards. All mice were individually housed on nonedible Aspen bedding in our GF-grade NesTiso caging system[12] and maintained on a 12-h:12-h light:dark cycle in a species-appropriate, temperature- and humidity-controlled room within AAALAC-accredited Animal Research Center facilities at Case Western Reserve University. All procedures were approved by the Institutional Animal Care and Use Committee and the Institutional Review Board at CWRU, in accordance with the Guide for Care and Use of Laboratory Animals.

B. Supplementary Discussion

Effect of the SoyP diet on fecal microbiome

While the effects of the SoyP diet appeared largely independent of baseline microbiome composition at the group level, microbiome structure shifted within individuals following dietary intake, indicating that both SoyP and AnimalP diets elicit modest individualized, rather than uniform, changes in microbial community composition. Notably, reductions in *Paraprevotella xylaniphila* (*Prevotellaceae* family), a species linked to CD through arabinoxylan and pectin metabolism and succinate production, were observed in the CD SoyP diet group. Although succinate has been associated with microbiome dysbiosis and intestinal inflammation[13, 14] its role in gut inflammatory processes remains incompletely defined.

Although *Enterobacteriaceae* were detected, they were not prominent in this dataset and were not among the taxa most influenced by diet. The increased abundance of *Streptococcaceae* observed among the CD AnimalP-diet group is of particular interest however, as the role of *Streptococcaceae* in CD remains poorly understood. The genus *Streptococcus* includes multiple species that differ in their pathogenic potential due to the presence of strain-specific toxins and virulence factors. Notably, alterations in *Streptococcus* abundance (*S. anginosus*) were also observed, albeit in the opposite direction in CD individuals consuming the SoyP-diet. This contrasting pattern suggests a diet-dependent modulation of *Streptococcus* populations, which may warrant further investigation in future studies.

Functional analysis (Lefse) revealed that, among CD participants, 7 days of the SoyP diet resulted in significant enrichment of two pathways with established anti-inflammatory relevance to CD, namely L-ornithine biosynthesis and the superpathway of geranylgeranyl diphosphate biosynthesis II (via the MEP pathway)[15, 16]. Microbiome-derived L-ornithine, produced by beneficial taxa such as *Faecalibacterium prausnitzii*, has been associated with enhanced efficacy of biologic therapies such as ustekinumab by disrupting IL-23/STAT3 signaling in immune cells, boosting treatment efficacy[17]. In this context, *F. prausnitzii* abundance increased in 37.5% (6/16) of CD participants consuming the SoyP-diet (paired t-test, $P = 0.33$). Enrichment of the geranylgeranyl diphosphate biosynthesis pathway has been associated with anti-inflammatory effects in experimental models of colitis, in part through its role in the maintenance and differentiation of regulatory T cells (Tregs)[16]. In parallel, the SoyP diet led to a significant decrease in 5-aminoimidazole ribonucleotide biosynthesis, a pathway which is part of the de novo purine synthesis pathway, shown to be enriched (upregulated) in CD inflammation[18-20]. Specifically, purine metabolism has been linked to intestinal permeability and regulating immune response in inflamed intestinal tissue (e.g., LC3/T helper type 17-interleukin 22 pathway)[21]. Collectively, these findings support the concept that a SoyP diet may serve to enhance or prolong remission response to traditional IBD therapies. By comparison, AnimalP intake led to an enrichment of peptidoglycan biosynthesis V (β-lactam resistance) with reductions in L-alanine biosynthesis, consistent with prior reports linking bacterial cell wall metabolism, NOD2 signaling, and immune activation to CD pathogenesis[22, 23]. Among HC participants, AnimalP intake was associated with enrichment of the GABA shunt and reductions in pathways related to L-glutamine biosynthesis. Although pathways such as the Calvin-Benson-

Bassham cycle and partial TCA variants do not directly correspond to human metabolism, they have been reported as microbial functional markers distinguishing health, remission, and therapeutic response states in IBD[24].

C. Supplementary Tables

Supplementary Table 1. Self-reported Foods Consumed by Participants on the Soy-pea Protein Diet that were Not Provided by the Study

Compliance N=16*	Foods
Compliant foods consumed that were not provided by the study, n=15 (94%)	fruit cup, mango juice, bell pepper, lemon, lime, Brussel sprouts, coconut, Brazil nuts, soy milk, romaine lettuce, mushrooms, pickles, hot sauce, apple, banana, celery, lime juice, rice vinegar, cupcake, granola, cookie, 5 cinnamon mints
Non-compliant foods consumed that were not provided by the study, n=1 (6%)	glass of skim milk

*A total of 16 of the 30 participants on the soy diet reported consuming foods that were not provided by the study, of whom 15 (50%) had foods that were still considered 'compliant' to the diet, and one participant reporting a single occurrence of consuming a non-compliant food.

Supplementary Table 2. Self-reported Foods Consumed by Participants on the Animal Protein Diet that were Not Provided by the Study

Compliance N=17*	Foods
Compliant foods consumed that were not provided by the study, n=17 (100%)	cheese, lemon, kiwi, apple, fruit cup, cucumber, avocado, broccoli sprouts, steak, chicken, broccoli, hamburger, lettuce, pickles, steak, soy free sauce, turkey bacon, butter, pepperoni, balsamic vinegar, stir fry veg, onions, celery, frozen veg, crackers, doughnut, breaded chicken wing, hard candy
Non-compliant foods consumed that were not provided by the study	n/a

*A total of 17 of the 30 participants on the animal protein diet reported consuming foods that were not provided by the study, of which all participants (100%) had foods that were still considered ‘compliant’ to the diet.

Supplementary Table 3. Within-group Total Daily Macronutrient and Fiber Intake Among Males

	Crohn's Disease SoyP (n=8)	Crohn's Disease AnimalP (n=5)	P-value*
Kcals	1894.8 (308.4)	1918.5 (443.8)	0.911
Fat (g)	90.7 (24.2)	108.6 (28.2)	0.248
Carb (g)	203 (48.3)	150.6 (48.8)	0.085
Pro (g)	102.8 (18.2)	105.3 (16.4)	0.808
Total Fiber	34.6 (8.1)	22.2 (6.7)	0.017
	**Healthy Control SoyP (n=3)**	**Healthy Control AnimalP (n=3)**	**P-value***
Kcals	2233.5 (828.5)	1745.5 (212.6)	0.183
Fat (g)	105.6 (57.6)	89.4 (14.9)	0.661
Carb (g)	276.5 (108)	171.2 (31.7)	0.181
Pro (g)	128.36 (40.88)	86.9 (13.1)	0.170
Total Fiber	50.3 (24.2)	19.6 (4.8)	0.098

AnimalP; animal protein diet, SoyP; soy-pea protein diet.

*Unpaired t-test p. Calculations include self-reported foods not provided by the study. Dietary intake data collected and analyzed using Nutrition Data System for Research software version 2019, developed by the Nutrition Coordinating Center (NCC), University of Minnesota, Minneapolis, MN. Results are mean, standard deviation.

Supplementary Table 4. Within-group Total Daily Macronutrient and Fiber Intake Among Females

	Crohn's Disease SoyP (n=8)	Crohn's Disease AnimalP (n=10)	P-value*
Kcals	1826.5 (246.8)	1761.7 (394.6)	0.691
Fat (g)	95.4 (24.4)	92.7 (26.2)	0.826
Carb (g)	199.4 (43.6)	167.2 (39.6)	0.121
Pro (g)	78.0 (8.9)	87.9 (19.9)	0.212
Total Fiber	31.1 (5.8)	26.9 (12.80)	0.415
	Healthy Control SoyP (n=11)	Healthy Control AnimalP (n=12)	P-value*
Kcals	1935.6 (691.0)	2050.8 (413.6)	0.629
Fat (g)	103.2 (43.6)	119.6 (29.1)	0.297
Carb (g)	205.1 (68.0)	171.2 (40.5)	0.157
Pro (g)	83.0 (30.1)	97.9 (23.1)	0.195
Total Fiber	35.1 (16.17)	26.9 (4.82)	0.109

AnimalP; animal protein diet, SoyP; soy-pea protein diet.

*Unpaired t-test p. Calculations include self-reported foods not provided by the study. Dietary intake data collected and analyzed using Nutrition Data System for Research software version 2019, developed by the Nutrition Coordinating Center (NCC), University of Minnesota, Minneapolis, MN. Results are mean, standard deviation.

Supplementary Table 5. Between-group Total Daily Macronutrient and Fiber Intake Among Males

	Healthy Control	Crohn's Disease	
	SoyP (n=3)	SoyP (n=8)	P-value*
Kcals	2233.5 (828.5)	1894.8 (308.4)	0.320
Fat (g)	105.6 (57.6)	90.7 (24.2)	0.661
Carb (g)	276.5 (108)	203 (48.3)	0.136
Pro (g)	128.36 (40.88)	102.8 (18.2)	0.166
Total Fiber	50.3 (24.2)	34.6 (8.1)	0.101
	AnimalP (n=3)	AnimalP (n=5)	P-value*
Kcals	1745.5 (212.6)	1918.5 (443.8)	0.558
Fat (g)	89.4 (14.9)	108.6 (28.2)	0.325
Carb (g)	171.2 (31.7)	150.6 (48.8)	0.543
Pro (g)	86.9 (13.1)	105.3 (16.4)	0.152
Total Fiber	19.6 (4.82)	22.2 (6.7)	0.601

AnimalP; animal protein diet, SoyP; soy-pea protein diet.

*Unpaired t-test p. Calculations include self-reported foods not provided by the study. Dietary intake data collected and analyzed using Nutrition Data System for Research software version 2019, developed by the Nutrition Coordinating Center (NCC), University of Minnesota, Minneapolis, MN. Results are mean, standard deviation.

Supplementary Table 6. Between-group Total Daily Macronutrient and Fiber Intake Among Females

	Healthy Control	Crohn's Disease	
	SoyP (n=11)	SoyP (n=8)	P-value*
Kcals	1935.6 (691.0)	1826.5 (246.8)	0.676
Fat (g)	103.2 (43.6)	95.4 (24.4)	0.655
Carb (g)	205.1 (68.0)	199.4 (43.6)	0.838
Pro (g)	83.0 (30.1)	78.0 (8.9)	0.656
Total Fiber	35.1 (16.1)	31.1 (5.8)	0.510
	AnimalP (n=12)	AnimalP (n=10)	P-value*
Kcals	2050.8 (413.6)	1761.7 (394.6)	0.111
Fat (g)	119.6 (29.1)	92.7 (26.2)	0.035
Carb (g)	171.2 (40.5)	167.2 (39.6)	0.818
Pro (g)	97.9 (23.1)	87.9 (19.9)	0.295
Total Fiber	26.9 (4.8)	26.9 (12.8)	0.939

AnimalP; animal protein diet, SoyP; soy-pea protein diet.

*Unpaired t-test p. Calculations include self-reported foods not provided by the study. Dietary intake data collected and analyzed using Nutrition Data System for Research software version 2019, developed by the Nutrition Coordinating Center (NCC), University of Minnesota, Minneapolis, MN. Results are mean, standard deviation.

Supplementary Table 7. Within-group Total Micronutrient Intake Over 7 Days Among Male Crohn's Disease Participants by Diet (SoyP vs AnimalP)

	SoyP (n=8)	AnimalP (n=5)	P-value*
Daidzein (mg)	332.95 (101.10)	0.69 (0.94)	0.00002
Genistein (mg)	428.83 (137.15)	1.29 (1.59)	0.00003
Glycitein (mg)	79.88 (26.43)	0.13 (0.26)	0.00004
Coumestrol (mg)	0.37 (0.38)	0.19 (0.23)	0.379
Biochanin A (mg)	0.74 (2.22)	2.44 (4.79)	0.399
Formononetin (mg)	0.07 (0.03)	0.06 (0.03)	0.648
Tryptophan (g)	7.73 (1.50)	7.70 (1.43)	0.973
Threonine (g)	22.65 (4.00)	26.51 (4.20)	0.124
Isoleucine (g)	26.38 (5.38)	29.46 (4.64)	0.313
Leucine (g)	44.74 (8.99)	49.33 (8.81)	0.387
Lysine (g)	33.97 (7.02)	43.69 (7.60)	0.038
Methionine (g)	8.18 (1.54)	14.66 (2.31)	0.0001
Cystine (g)	7.98 (1.41)	8.28 (2.04)	0.764
Phenylalanine (g)	29.91 (6.00)	28.26 (5.29)	0.624
Tyrosine (g)	20.69 (4.10)	22.94 (3.31)	0.327
Valine (g)	28.67 (5.28)	33.32 (6.39)	0.180
Arginine (g)	49.87 (9.52)	40.99 (12.03)	0.166
Histidine (g)	15.58 (3.13)	17.42 (3.15)	0.325
Alanine (g)	26.27 (4.79)	30.60 (5.86)	0.172
Aspartic Acid (g)	70.80 (14.09)	60.57 (11.28)	0.199
Glutamic Acid (g)	121.34 (21.95)	113.88 (23.57)	0.573
Glycine (g)	26.99 (4.84)	27.80 (6.90)	0.806
Proline (g)	31.79 (6.65)	35.75 (6.17)	0.306
Serine (g)	31.95 (6.58)	29.53 (6.05)	0.521
Vit A (IU)	64184.54 (31563.76)	101527.99 (25105.71)	0.048
Vit D (mcg)	29.55 (8.54)	24.86 (9.41)	0.374
Vit E (mg)	68.64 (22.18)	78.11 (29.66)	0.523
Vit K (mcg)	1680.65 (622.16)	1581.29 (299.90)	0.748
Vit C (mg)	1215.37 (646.61)	929.65 (310.65)	0.381
Thiamin (mg)	8.92 (2.43)	5.45 (2.40)	0.029
Riboflavin (mg)	11.12 (2.09)	11.57 (1.65)	0.691
Niacin (mg)	94.01 (35.69)	152.32 (42.68)	0.022
Pantothenic Acid (mg)	28.95 (10.22)	40.14 (9.37)	0.073
Vit B-6 (mg)	12.37 (3.21)	14.34 (3.86)	0.338
Folate (mcg)	3710.79 (83.53)	2078.02 (355.06)	<0.000001
B- 12 (mcg)	3710.79 (8.38)	29.71 (6.10)	<0.000001
Calcium (mg)	6351.26 (1053.21)	6709.49 (1248.65)	0.589
Phosphorous (mg)	9198.60 (1622.75)	9564.23 (2398.35)	0.747
Magnesium (mg)	3430.84 (861.19)	2494.85 (912.66)	0.089
Iron (mg)	135.19 (24.01)	77.29 (28.74)	0.002
Zinc (mg)	65.87 (15.39)	83.77 (28.15)	0.162
Copper (mg)	23.14 (22.74)	11.98 (6.74)	0.315
Selenium (mcg)	244.48 (71.65)	615.81 (156.47)	0.0001
Sodium (mg)	16647.12 (9780.58)	17421.38 (5389.14)	0.875
Potassium (mg)	28267.74 (5368.04)	21801.23 (5726.76)	0.064

AnimalP; animal protein diet, SoyP; soy-pea protein diet.

*Unpaired t-test p. Calculations include self-reported foods not provided by the study. Dietary intake data collected and analyzed using Nutrition Data System for Research software version 2019, developed by the Nutrition Coordinating Center (NCC), University of Minnesota, Minneapolis, MN. Results are mean, standard deviation.

Supplementary Table 8. Within-group Total Micronutrient Intake Over 7 Days Among Male Healthy Control Participants by Diet (SoyP vs AnimalP)

	SoyP (n=3)	AnimalP (n=3)	P-value*
Daidzein (mg)	331.28 (87.95)	0.12 (0.07)	0.003
Genistein (mg)	434.28 (115.04)	0.72 (0.68)	0.003
Glycitein (mg)	66.63 (17.11)	0.00 (0.00)	0.003
Coumestrol (mg)	0.26 (0.23)	0.04 (0.07)	0.178
Biochanin A (mg)	0.05 (0.01)	0.02 (0.02)	0.081
Formononetin (mg)	0.14 (0.02)	0.04 (0.02)	0.002
Tryptophan (g)	9.64 (2.86)	6.39 (1.25)	0.146
Threonine (g)	27.87 (8.54)	21.05 (4.04)	0.280
Isoleucine (g)	33.08 (10.12)	23.83 (4.67)	0.224
Leucine (g)	55.87 (17.30)	40.58 (7.25)	0.231
Lysine (g)	41.10 (12.59)	35.96 (7.00)	0.569
Methionine (g)	10.56 (3.10)	11.63 (2.39)	0.659
Cystine (g)	10.53 (3.09)	6.71 (0.90)	0.109
Phenylalanine (g)	38.12 (12.23)	23.28 (4.56)	0.120
Tyrosine (g)	25.11 (7.87)	18.10 (4.27)	0.247
Valine (g)	36.56 (10.99)	27.03 (5.28)	0.248
Arginine (g)	64.47 (20.33)	36.42 (4.42)	0.080
Histidine (g)	19.66 (6.32)	14.52 (2.36)	0.257
Alanine (g)	33.12 (10.61)	25.24 (4.15)	0.297
Aspartic Acid (g)	88.42 (29.18)	52.08 (10.84)	0.113
Glutamic Acid (g)	156.08 (49.12)	97.25 (18.35)	0.124
Glycine (g)	35.79 (11.99)	22.99 (2.68)	0.145
Proline (g)	38.69 (12.41)	27.49 (7.16)	0.247
Serine (g)	39.52 (12.28)	24.26 (4.65)	0.114
Vit A (IU)	46437.68 (17985.98)	25606.07 (20788.15)	0.260
Vit D (mcg)	32.09 (7.29)	39.33 (51.87)	0.823
Vit E (mg)	175.24 (116.50)	73.31 (18.16)	0.209
Vit K (mcg)	2078.73 (1405.88)	1147.01 (1114.83)	0.419
Vit C (mg)	1520.68 (786.49)	873.48 (873.09)	0.394
Thiamin (mg)	9.85 (3.01)	4.61 (0.77)	0.043
Riboflavin (mg)	13.23 (5.75)	9.65 (3.67)	0.416
Niacin (mg)	124.37 (50.67)	127.40 (37.03)	0.937
Pantothenic Acid (mg)	48.12 (20.12)	33.16 (12.99)	0.340
Vit B-6 (mg)	18.04 (6.39)	12.27 (3.86)	0.251
Folate (mcg)	4160.44 (1649.38)	1520.37 (1003.23)	0.077
B- 12 (mcg)	24.93 (5.44)	20.10 (7.79)	0.428
Calcium (mg)	7095.85 (3053.84)	4054.93 (1727.81)	0.208
Phosphorous (mg)	13327.84 (3774.09)	8144.98 (1688.50)	0.096
Magnesium (mg)	4741.40 (1567.74)	2496.95 (237.06)	0.070
Iron (mg)	167.75 (54.40)	63.10 (5.24)	0.029
Zinc (mg)	94.80 (27.10)	65.57 (11.15)	0.159
Copper (mg)	32.94 (9.12)	11.87 (1.16)	0.017
Selenium (mcg)	410.19 (78.99)	494.54 (132.81)	0.398
Sodium (mg)	15073.14 (7546.94)	12668.31 (132.81)	0.610
Potassium (mg)	36315.79 (14003.99)	18735.86 (5889.65)	0.116

AnimalP; animal protein diet, SoyP; soy-pea protein diet.

*Unpaired t-test p. Calculations include self-reported foods not provided by the study. Dietary intake data collected and analyzed using Nutrition Data System for Research software version 2019, developed by the Nutrition Coordinating Center (NCC), University of Minnesota, Minneapolis, MN. Results are mean, standard deviation.

Supplementary Table 9. Within-group Total Micronutrient Intake Over 7 Days Among Female Crohn's Disease Participants by Diet (SoyP vs AnimalP)

	SoyP (n=8)	AnimalP (n=10)	P-value*
Daidzein (mg)	232.75 (65.35)	1.61 (4.73)	<0.000001
Genistein (mg)	289.16 (78.18)	2.75 (6.64)	<0.000001
Glycitein (mg)	59.09 (14.07)	0.35 (1.09)	<0.000001
Coumestrol (mg)	0.44 (0.34)	0.07 (0.12)	0.005
Biochanin A (mg)	0.06 (0.07)	0.02 (0.02)	0.175
Formononetin (mg)	0.10 (0.08)	0.05 (0.04)	0.087
Tryptophan (g)	5.68 (0.84)	6.29 (1.60)	0.344
Threonine (g)	17.11 (2.33)	21.20 (5.04)	0.050
Isoleucine (g)	19.21 (2.96)	24.03 (6.33)	0.065
Leucine (g)	19.21 (4.73)	41.07 (10.17)	0.00004
Lysine (g)	24.36 (3.20)	36.24 (8.87)	0.002
Methionine (g)	6.12 (0.94)	36.24 (3.01)	<0.000001
Cystine (g)	6.06 (0.92)	36.24 (1.81)	<0.000001
Phenylalanine (g)	22.69 (0.34)	23.77 (6.38)	0.640
Tyrosine (g)	15.33 (1.95)	23.77 (5.12)	0.0005
Valine (g)	21.39 (3.00)	27.72 (7.45)	0.039
Arginine (g)	39.69 (5.61)	34.63 (10.10)	0.224
Histidine (g)	11.83 (1.49)	14.90 (3.73)	0.044
Alanine (g)	20.15 (2.62)	25.24 (6.30)	0.049
Aspartic Acid (g)	54.42 (6.61)	51.32 (13.24)	0.555
Glutamic Acid (g)	94.96 (13.19)	96.33 (23.77)	0.886
Glycine (g)	21.63 (2.96)	23.22 (6.50)	0.531
Proline (g)	23.59 (3.53)	30.78 (8.03)	0.032
Serine (g)	24.05 (3.18)	24.79 (6.48)	0.772
Vit A (IU)	65145.57 (52687.07)	66957.41 (29239.34)	0.927
Vit D (mcg)	20.13 (8.04)	16.22 (9.64)	0.372
Vit E (mg)	99.70 (66.31)	82.05 (50.63)	0.530
Vit K (mcg)	1317.99 (542.40)	1099.86 (602.34)	0.437
Vit C (mg)	859.50 (574.11)	789.11 (458.27)	0.776
Thiamin (mg)	6.74 (1.61)	4.52 (1.25)	0.004
Riboflavin (mg)	8.52 (1.52)	10.06 (2.80)	0.184
Niacin (mg)	90.55 (16.92)	124.14 (49.02)	0.084
Pantothenic Acid (mg)	26.30 (9.68)	34.94 (13.56)	0.149
Vit B-6 (mg)	11.23 (2.59)	13.26 (3.64)	0.204
Folate (mcg)	3237.96 (929.21)	1904.89 (696.20)	0.003
B- 12 (mcg)	12.19 (7.59)	23.08 (6.35)	0.004
Calcium (mg)	4963.73 (867.80)	5111.39 (2089.58)	0.854
Phosphorous (mg)	7874.46 (1576.87)	8437.84 (2631.57)	0.602
Magnesium (mg)	3198.45 (514.45)	2362.23 (717.41)	0.014
Iron (mg)	104.39 (16.79)	66.73 (20.76)	0.001
Zinc (mg)	57.41 (10.67)	74.52 (19.37)	0.040
Copper (mg)	19.64 (4.29)	11.46 (3.81)	0.001
Selenium (mcg)	237.05 (89.17)	520.06 (187.76)	0.001
Sodium (mg)	13042.21 (5616.16)	12510.68 (5090.05)	0.836
Potassium (mg)	24195.35 (3951.84)	20274.61 (5220.05)	0.098

AnimalP; animal protein diet, SoyP; soy-pea protein diet.

*Unpaired t-test p. Calculations include self-reported foods not provided by the study. Dietary intake data collected and analyzed using Nutrition Data System for Research software version 2019, developed by the Nutrition Coordinating Center (NCC), University of Minnesota, Minneapolis, MN. Results are mean, standard deviation.

Supplementary Table 10. Within-group Total Micronutrient Intake Over 7 Days Among Female Healthy Control Participants by Diet (SoyP vs AnimalP)

	SoyP (n=11)	AnimalP (n=12)	P-value*
Daidzein (mg)	255.34 (86.37)	0.15 (0.08)	<0.000001
Genistein (mg)	324.06 (111.44)	0.62 (0.58)	<0.000001
Glycitein (mg)	54.27 (1.60)	0.00 (0.00)	<0.000001
Coumestrol (mg)	0.34 (0.18)	0.10 (0.14)	0.002
Biochanin A (mg)	0.02 (0.01)	1.19 (3.86)	0.327
Formononetin (mg)	0.06 (0.04)	0.06 (0.05)	0.697
Tryptophan (g)	6.15 (2.38)	7.21 (1.76)	0.238
Threonine (g)	6.15 (6.89)	23.45 (5.57)	0.000001
Isoleucine (g)	21.02 (8.29)	25.94 (6.72)	0.131
Leucine (g)	35.84 (13.72)	45.40 (11.18)	0.080
Lysine (g)	26.61 (9.89)	39.91 (9.93)	0.004
Methionine (g)	6.60 (2.67)	13.00 (3.15)	0.000
Cystine (g)	6.60 (2.57)	7.02 (1.59)	0.635
Phenylalanine (g)	24.25 (9.48)	26.01 (6.56)	0.608
Tyrosine (g)	24.25 (6.15)	20.81 (5.43)	0.169
Valine (g)	23.15 (9.07)	29.96 (7.43)	0.061
Arginine (g)	40.61 (16.17)	37.79 (9.92)	0.615
Histidine (g)	12.60 (4.88)	16.20 (4.13)	0.069
Alanine (g)	21.49 (8.32)	27.66 (6.32)	0.057
Aspartic Acid (g)	57.79 (21.98)	57.95 (13.84)	0.984
Glutamic Acid (g)	99.31 (38.39)	108.71 (27.43)	0.503
Glycine (g)	22.64 (9.17)	24.93 (6.30)	0.490
Proline (g)	25.10 (9.48)	34.33 (9.81)	0.033
Serine (g)	25.44 (9.63)	27.52 (6.74)	0.551
Vit A (IU)	41217.13 (29235.27)	55278.43 (27674.21)	0.249
Vit D (mcg)	24.88 (11.11)	42.35 (30.85)	0.091
Vit E (mg)	112.45 (967.55)	119.40 (32.59)	0.980
Vit K (mcg)	1519.94 (914.91)	1737.03 (738.63)	0.536
Vit C (mg)	943.83 (530.10)	1030.50 (425.22)	0.668
Thiamin (mg)	7.55 (2.78)	5.18 (1.68)	0.021
Riboflavin (mg)	10.30 (3.60)	12.08 (3.41)	0.237
Niacin (mg)	83.14 (40.75)	135.18 (44.60)	0.008
Pantothenic Acid (mg)	30.15 (17.59)	40.33 (7.65)	0.082
Vit B-6 (mg)	11.77 (6.90)	13.59 (2.88)	0.411
Folate (mcg)	2993.92 (1203.95)	2096.34 (606.86)	0.033
B- 12 (mcg)	20.63 (12.43)	26.27 (7.52)	0.197
Calcium (mg)	5577.14 (2026.25)	6189.01 (1603.56)	0.429
Phosphorous (mg)	7980.69 (3484.80)	9613.98 (2444.03)	0.204
Magnesium (mg)	3208.01 (1296.63)	2679.87 (680.01)	0.229
Iron (mg)	115.64 (47.50)	69.47 (16.04)	0.004
Zinc (mg)	61.97 (32.24)	76.76 (21.62)	0.207
Copper (mg)	20.92 (8.24)	13.00 (3.06)	0.005
Selenium (mcg)	237.79 (116.99)	537.01 (135.58)	0.00001
Sodium (mg)	13167.80 (4756.00)	14578.50 (3081.55)	0.404
Potassium (mg)	25520.77 (9487.25)	22884.26 (4832.22)	0.404

AnimalP; animal protein diet, SoyP; soy-pea protein diet.

*Unpaired t-test p. Calculations include self-reported foods not provided by the study. Dietary intake data collected and analyzed using Nutrition Data System for Research software version 2019, developed by the Nutrition Coordinating Center (NCC), University of Minnesota, Minneapolis, MN. Results are mean, standard deviation.

Supplementary Table 11. Between-group Total Micronutrient Intake Over 7 Days Among Male Crohn's Disease and Healthy Control Participants Assigned the SoyP diet

	Healthy Control (n=3)	Crohn's Disease (n=8)	P-value*
Daidzein (mg)	331.28 (87.95)	332.95 (101.10)	0.981
Genistein (mg)	434.28 (115.04)	428.83 (137.15)	0.953
Glycitein (mg)	66.63 (17.11)	79.88 (26.43)	0.448
Coumestrol (mg)	0.26 (0.23)	0.37 (0.38)	0.669
Biochanin A (mg)	0.05 (0.01)	0.74 (2.22)	0.612
Formononetin (mg)	0.14 (0.02)	0.07 (0.03)	0.003
Tryptophan (g)	9.64 (2.86)	7.73 (1.50)	0.169
Threonine (g)	27.87 (8.54)	22.65 (4.00)	0.184
Isoleucine (g)	33.08 (10.12)	26.38 (5.38)	0.175
Leucine (g)	55.87 (17.30)	44.74 (8.99)	0.182
Lysine (g)	41.10 (12.59)	33.97 (7.02)	0.250
Methionine (g)	10.56 (3.10)	8.18 (1.54)	0.112
Cystine (g)	10.53 (3.09)	7.98 (1.41)	0.081
Phenylalanine (g)	38.12 (12.23)	29.91 (6.00)	0.156
Tyrosine (g)	25.11 (7.87)	20.69 (4.10)	0.240
Valine (g)	36.56 (10.99)	28.67 (5.28)	0.129
Arginine (g)	64.47 (20.33)	49.87 (9.52)	0.125
Histidine (g)	19.66 (6.32)	15.58 (3.13)	0.171
Alanine (g)	33.12 (10.61)	26.27 (4.79)	0.157
Aspartic Acid (g)	88.42 (29.18)	70.80 (14.09)	0.194
Glutamic Acid (g)	156.08 (49.12)	121.34 (21.95)	0.123
Glycine (g)	35.79 (11.99)	26.99 (4.84)	0.099
Proline (g)	38.69 (12.41)	31.79 (6.65)	0.249
Serine (g)	39.52 (12.28)	31.95 (6.58)	0.205
Vit A (IU)	46437.68 (17985.98)	64184.54 (31563.76)	0.391
Vit D (mcg)	32.09 (7.29)	29.55 (8.54)	0.661
Vit E (mg)	175.24 (116.50)	68.64 (22.18)	0.024
Vit K (mcg)	2078.73 (1405.88)	1680.65 (622.16)	0.512
Vit C (mg)	1520.68 (786.49)	1215.37 (646.61)	0.524
Thiamin (mg)	9.85 (3.01)	8.92 (2.43)	0.604
Riboflavin (mg)	13.23 (5.75)	11.12 (2.09)	0.368
Niacin (mg)	124.37 (50.67)	94.01 (35.69)	0.286
Pantothenic Acid (mg)	48.12 (20.12)	28.95 (10.22)	0.059
Vit B-6 (mg)	18.04 (6.39)	12.37 (3.21)	0.073
Folate (mcg)	4160.44 (1649.38)	3710.79 (83.53)	0.417
B- 12 (mcg)	24.93 (5.44)	3710.79 (8.38)	<0.000001
Calcium (mg)	7095.85 (3053.84)	6351.26 (1053.21)	0.537
Phosphorous (mg)	13327.84 (3774.09)	9198.60 (1622.75)	0.026
Magnesium (mg)	4741.40 (1567.74)	3430.84 (861.19)	0.101
Iron (mg)	167.75 (54.40)	135.19 (24.01)	0.182
Zinc (mg)	94.80 (27.10)	65.87 (15.39)	0.048
Copper (mg)	32.94 (9.12)	23.14 (22.74)	0.498
Selenium (mcg)	410.19 (78.99)	244.48 (71.65)	0.009
Sodium (mg)	15073.14 (7546.94)	16647.12 (9780.58)	0.809
Potassium (mg)	36315.79 (14003.99)	28267.74 (5368.04)	0.177

AnimalP; animal protein diet, SoyP; soy-pea protein diet.

*Unpaired t-test p. Calculations include self-reported foods not provided by the study. Dietary intake data collected and analyzed using Nutrition Data System for Research software version 2019, developed by the Nutrition Coordinating Center (NCC), University of Minnesota, Minneapolis, MN. Results are mean, standard deviation.

Supplementary Table 12. Between-group Total Micronutrient Intake Over 7 Days Among Male Crohn's Disease and Healthy Control Participants Assigned the AnimalP diet

	Healthy Control (n=3)	Crohn's Disease (n=5)	P-value*
Kcals	1745.50 (212.60)	1745.50 (212.60)	0.558
Fat (g)	89.40 (14.90)	89.40 (14.90)	0.325
Carb (g)	171.20 (31.70)	171.20 (31.70)	0.543
Pro (g)	86.90 (13.10)	86.90 (13.10)	0.152
Total Fiber	137.80 (33.90)	137.80 (33.90)	0.601
Daidzein (mg)	0.12 (0.07)	0.12 (0.07)	0.350
Genistein (mg)	0.72 (0.68)	0.72 (0.68)	0.589
Glycitein (mg)	0.00 (0.00)	0.00 (0.00)	0.434
Coumestrol (mg)	0.04 (0.07)	0.04 (0.07)	0.323
Biochanin A (mg)	0.02 (0.02)	0.02 (0.02)	0.430
Formononetin (mg)	0.04 (0.02)	0.04 (0.02)	0.449
Tryptophan (g)	6.39 (1.25)	6.39 (1.25)	0.241
Threonine (g)	21.05 (4.04)	21.05 (4.04)	0.121
Isoleucine (g)	23.83 (4.67)	23.83 (4.67)	0.148
Leucine (g)	40.58 (7.25)	40.58 (7.25)	0.200
Lysine (g)	35.96 (7.00)	35.96 (7.00)	0.202
Methionine (g)	11.63 (2.39)	11.63 (2.39)	0.127
Cystine (g)	6.71 (0.90)	6.71 (0.90)	0.264
Phenylalanine (g)	23.28 (4.56)	23.28 (4.56)	0.226
Tyrosine (g)	18.10 (4.27)	18.10 (4.27)	0.120
Valine (g)	27.03 (5.28)	27.03 (5.28)	0.204
Arginine (g)	36.42 (4.42)	36.42 (4.42)	0.560
Histidine (g)	14.52 (2.36)	14.52 (2.36)	0.222
Alanine (g)	25.24 (4.15)	25.24 (4.15)	0.219
Aspartic Acid (g)	52.08 (10.84)	52.08 (10.84)	0.337
Glutamic Acid (g)	97.25 (18.35)	97.25 (18.35)	0.340
Glycine (g)	22.99 (2.68)	22.99 (2.68)	0.302
Proline (g)	27.49 (7.16)	27.49 (7.16)	0.133
Serine (g)	24.26 (4.65)	24.26 (4.65)	0.246
Vit A (IU)	25606.07 (20788.15)	25606.07 (20788.15)	0.005
Vit D (mcg)	39.33 (51.87)	39.33 (51.87)	0.545
Vit E (mg)	73.31 (18.16)	73.31 (18.16)	0.812
Vit K (mcg)	1147.01 (1114.83)	1147.01 (1114.83)	0.421
Vit C (mg)	873.48 (873.09)	873.48 (873.09)	0.896
Thiamin (mg)	4.61 (0.77)	4.61 (0.77)	0.589
Riboflavin (mg)	9.65 (3.67)	9.65 (3.67)	0.335
Niacin (mg)	127.40 (37.03)	127.40 (37.03)	0.436
Pantothenic Acid (mg)	33.16 (12.99)	33.16 (12.99)	0.407
Vit B-6 (mg)	12.27 (3.86)	12.27 (3.86)	0.490
Folate (mcg)	1520.37 (1003.23)	1520.37 (1003.23)	0.283
B- 12 (mcg)	20.10 (7.79)	20.10 (7.79)	0.098
Calcium (mg)	4054.93 (1727.81)	4054.93 (1727.81)	0.044
Phosphorous (mg)	8144.98 (1688.50)	8144.98 (1688.50)	0.409
Magnesium (mg)	2496.95 (237.06)	2496.95 (237.06)	0.997
Iron (mg)	63.10 (5.24)	63.10 (5.24)	0.443
Zinc (mg)	65.57 (11.15)	65.57 (11.15)	0.337
Copper (mg)	11.87 (1.16)	11.87 (1.16)	0.978
Selenium (mcg)	494.54 (132.81)	494.54 (132.81)	0.308
Sodium (mg)	12668.31 (132.81)	12668.31 (132.81)	0.190
Potassium (mg)	18735.86 (5889.65)	18735.86 (5889.65)	0.495

AnimalP; animal protein diet, SoyP; soy-pea protein diet.

*Unpaired t-test p. Calculations include self-reported foods not provided by the study. Dietary intake data collected and analyzed using Nutrition Data System for Research software version 2019, developed by the Nutrition Coordinating Center (NCC), University of Minnesota, Minneapolis, MN. Results are mean, standard deviation.

Supplementary Table 13. Between-group Total Micronutrient Intake Over 7 Days Among Female Crohn's Disease and Healthy Control Participants Assigned the SoyP diet

	Healthy Control (n=11)	Crohn's Disease (n=8)	P-value*
Daidzein (mg)	255.34 (86.37)	232.75 (65.35)	0.543
Genistein (mg)	324.06 (111.44)	289.16 (78.18)	0.459
Glycitein (mg)	54.27 (1.60)	59.09 (14.07)	0.271
Coumestrol (mg)	0.34 (0.18)	0.44 (0.34)	0.420
Biochanin A (mg)	0.02 (0.01)	0.06 (0.07)	0.115
Formononetin (mg)	0.06 (0.04)	0.10 (0.08)	0.212
Tryptophan (g)	6.15 (2.38)	5.68 (0.84)	0.600
Threonine (g)	6.15 (6.89)	17.11 (2.33)	0.0005
Isoleucine (g)	21.02 (8.29)	19.21 (2.96)	0.564
Leucine (g)	35.84 (13.72)	19.21 (4.73)	0.005
Lysine (g)	26.61 (9.89)	24.36 (3.20)	0.546
Methionine (g)	6.60 (2.67)	6.12 (0.94)	0.636
Cystine (g)	6.60 (2.57)	6.06 (0.92)	0.580
Phenylalanine (g)	24.25 (9.48)	22.69 (0.34)	0.650
Tyrosine (g)	24.25 (6.15)	15.33 (1.95)	0.001
Valine (g)	23.15 (9.07)	21.39 (3.00)	0.606
Arginine (g)	40.61 (16.17)	39.69 (5.61)	0.880
Histidine (g)	12.60 (4.88)	11.83 (1.49)	0.673
Alanine (g)	21.49 (8.32)	20.15 (2.62)	0.669
Aspartic Acid (g)	57.79 (21.98)	54.42 (6.61)	0.682
Glutamic Acid (g)	99.31 (38.39)	94.96 (13.19)	0.764
Glycine (g)	22.64 (9.17)	21.63 (2.96)	0.767
Proline (g)	25.10 (9.48)	23.59 (3.53)	0.673
Serine (g)	25.44 (9.63)	24.05 (3.18)	0.701
Vit A (IU)	41217.13 (29235.27)	65145.57 (52687.07)	0.221
Vit D (mcg)	24.88 (11.11)	20.13 (8.04)	0.319
Vit E (mg)	112.45 (967.55)	99.70 (66.31)	0.971
Vit K (mcg)	1519.94 (914.91)	1317.99 (542.40)	0.586
Vit C (mg)	943.83 (530.10)	859.50 (574.11)	0.745
Thiamin (mg)	7.55 (2.78)	6.74 (1.61)	0.472
Riboflavin (mg)	10.30 (3.60)	8.52 (1.52)	0.208
Niacin (mg)	83.14 (40.75)	90.55 (16.92)	0.636
Pantothenic Acid (mg)	30.15 (17.59)	26.30 (9.68)	0.584
Vit B-6 (mg)	11.77 (6.90)	11.23 (2.59)	0.838
Folate (mcg)	2993.92 (1203.95)	3237.96 (929.21)	0.639
B- 12 (mcg)	20.63 (12.43)	12.19 (7.59)	0.108
Calcium (mg)	5577.14 (2026.25)	4963.73 (867.80)	0.435
Phosphorous (mg)	7980.69 (3484.80)	7874.46 (1576.87)	0.937
Magnesium (mg)	3208.01 (1296.63)	3198.45 (514.45)	0.985
Iron (mg)	115.64 (47.50)	104.39 (16.79)	0.532
Zinc (mg)	61.97 (32.24)	57.41 (10.67)	0.707
Copper (mg)	20.92 (8.24)	19.64 (4.29)	0.696
Selenium (mcg)	237.79 (116.99)	237.05 (89.17)	0.988
Sodium (mg)	13167.80 (4756.00)	13042.21 (5616.16)	0.959
Potassium (mg)	25520.77 (9487.25)	24195.35 (3951.84)	0.716

AnimalP; animal protein diet, SoyP; soy-pea protein diet.

*Unpaired t-test p. Calculations include self-reported foods not provided by the study. Dietary intake data collected and analyzed using Nutrition Data System for Research software version 2019, developed by the Nutrition Coordinating Center (NCC), University of Minnesota, Minneapolis, MN. Results are mean, standard deviation.

Supplementary Table 14. Between-group Total Micronutrient Intake Over 7 Days Among Female Crohn's Disease and Healthy Control Participants Assigned the AnimalP diet

	Healthy Control (n=12)	Crohn's Disease (n=10)	P-value*
Daidzein (mg)	0.15 (0.08)	1.61 (4.73)	0.294
Genistein (mg)	0.62 (0.58)	2.75 (6.64)	0.279
Glycitein (mg)	0.00 (0.00)	0.35 (1.09)	0.284
Coumestrol (mg)	0.10 (0.14)	0.07 (0.12)	0.564
Biochanin A (mg)	1.19 (3.86)	0.02 (0.02)	0.352
Formononetin (mg)	0.06 (0.05)	0.05 (0.04)	0.632
Tryptophan (g)	7.21 (1.76)	6.29 (1.60)	0.222
Threonine (g)	23.45 (5.57)	21.20 (5.04)	0.335
Isoleucine (g)	25.94 (6.72)	24.03 (6.33)	0.503
Leucine (g)	45.40 (11.18)	41.07 (10.17)	0.358
Lysine (g)	39.91 (9.93)	36.24 (8.87)	0.376
Methionine (g)	13.00 (3.15)	36.24 (3.01)	<0.000001
Cystine (g)	7.02 (1.59)	36.24 (1.81)	<0.000001
Phenylalanine (g)	26.01 (6.56)	23.77 (6.38)	0.429
Tyrosine (g)	20.81 (5.43)	23.77 (5.12)	0.206
Valine (g)	29.96 (7.43)	27.72 (7.45)	0.489
Arginine (g)	37.79 (9.92)	34.63 (10.10)	0.470
Histidine (g)	16.20 (4.13)	14.90 (3.73)	0.452
Alanine (g)	27.66 (6.32)	25.24 (6.30)	0.381
Aspartic Acid (g)	57.95 (13.84)	51.32 (13.24)	0.268
Glutamic Acid (g)	108.71 (27.43)	96.33 (23.77)	0.276
Glycine (g)	24.93 (6.30)	23.22 (6.50)	0.538
Proline (g)	34.33 (9.81)	30.78 (8.03)	0.371
Serine (g)	27.52 (6.74)	24.79 (6.48)	0.346
Vit A (IU)	55278.43 (27674.21)	66957.41 (29239.34)	0.348
Vit D (mcg)	42.35 (30.85)	16.22 (9.64)	0.018
Vit E (mg)	119.40 (32.59)	82.05 (50.63)	0.049
Vit K (mcg)	1737.03 (738.63)	1099.86 (602.34)	0.041
Vit C (mg)	1030.50 (425.22)	789.11 (458.27)	0.215
Thiamin (mg)	5.18 (1.68)	4.52 (1.25)	0.312
Riboflavin (mg)	12.08 (3.41)	10.06 (2.80)	0.149
Niacin (mg)	135.18 (44.60)	124.14 (49.02)	0.587
Pantothenic Acid (mg)	40.33 (7.65)	34.94 (13.56)	0.254
Vit B-6 (mg)	13.59 (2.88)	13.26 (3.64)	0.815
Folate (mcg)	2096.34 (606.86)	1904.89 (696.20)	0.498
B- 12 (mcg)	26.27 (7.52)	23.08 (6.35)	0.301
Calcium (mg)	6189.01 (1603.56)	5111.39 (2089.58)	0.186
Phosphorous (mg)	9613.98 (2444.03)	8437.84 (2631.57)	0.291
Magnesium (mg)	2679.87 (680.01)	2362.23 (717.41)	0.300
Iron (mg)	69.47 (16.04)	66.73 (20.76)	0.730
Zinc (mg)	76.76 (21.62)	74.52 (19.37)	0.802
Copper (mg)	13.00 (3.06)	11.46 (3.81)	0.307
Selenium (mcg)	537.01 (135.58)	520.06 (187.76)	0.808
Sodium (mg)	14578.50 (3081.55)	12510.68 (5090.05)	0.254
Potassium (mg)	22884.26 (4832.22)	20274.61 (5220.05)	0.238

AnimalP; animal protein diet, SoyP; soy-pea protein diet.

*Unpaired t-test p. Calculations include self-reported foods not provided by the study. Dietary intake data collected and analyzed using Nutrition Data System for Research software version 2019, developed by the Nutrition Coordinating Center (NCC), University of Minnesota, Minneapolis, MN. Results are mean, standard deviation.

D. Supplementary Figure

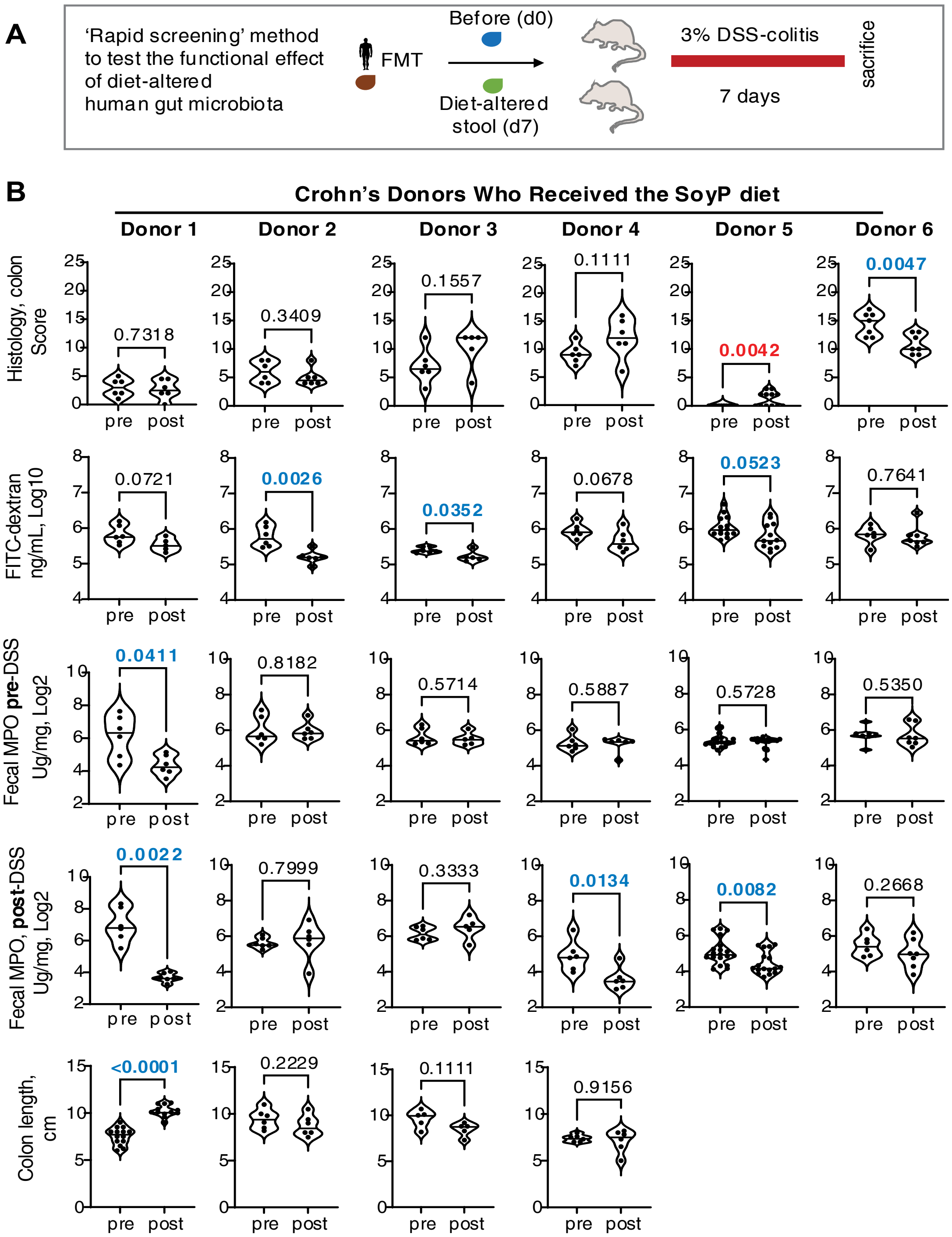

Supplementary Figure 2. Fecal microbiota transplantation experiments using baseline ("pre") and diet-altered ("post") stool collected from Crohn's disease participants assigned to the SoyP-diet intervention. A) Study Design for Fecal Microbiota Transplantation Experiments. Germ free SAMP mice transplanted with baseline ('pre'; d0) or diet-altered stool ('post'; d7) and then treated with 3% DSS for 7 days (n=6 mice/group). **B)** Colitis severity indices including histology, gut permeability (FITC-dextran), fecal myeloperoxidase (MPO), and colon length. Gut permeability and fecal MPO were measured *in vivo* before and after DSS induction. Colon length data missing for donor 5 and 6. Significant p-values where an improvement was observed indicated in bold blue font, and p-values where worsening was observed indicated in bold red font.

E. Supplementary References

1. Rodriguez-Palacios A, Harding A, Menghini P, et al. The Artificial Sweetener Splenda Promotes Gut Proteobacteria, Dysbiosis, and Myeloperoxidase Reactivity in Crohn's Disease-Like Ileitis. Inflammatory Bowel Diseases 2018;24:1005-1020.
2. Rodriguez-Palacios A, Kodani T, Kaydo L, et al. Stereomicroscopic 3D-pattern profiling of murine and human intestinal inflammation reveals unique structural phenotypes. Nat Commun 2015;6:7577.
3. Rodriguez-Palacios A, Aladyshkina N, Cominelli F. Stereomicroscopy and 3D-target myeloperoxidase intestinal phenotyping following a fecal flora homogenization protocol. Protocol Exchange 2015.
4. Basson AR, Gomez-Nguyen A, Menghini P, et al. Human Gut Microbiome Transplantation in Ileitis Prone Mice: A Tool for the Functional Characterization of the Microbiota in Inflammatory Bowel Disease Patients. Inflamm Bowel Dis 2020;26:347-359.
5. Basson A, Gomez-Nguyen A, LaSalla A, et al. Replacing Animal Protein with Soy-Pea Protein in an ‘American diet’ Controls Murine Crohn’s Disease-Like Ileitis Regardless of Firmicutes:Bacteroidetes ratio. Journal of Nutrition 9999;151:579-590.
6. Prosberg M, Bendtsen F, Vind I, et al. The association between the gut microbiota and the inflammatory bowel disease activity: a systematic review and meta-analysis. Scand J Gastroenterol 2016;51:1407-1415.
7. Menghini P, Corridoni D, Buttó LF, et al. Neutralization of IL-1α ameliorates Crohn's disease-like ileitis by functional alterations of the gut microbiome. Proc Natl Acad Sci U S A 2019.
8. Thevaranjan N, Puchta A, Schulz C, et al. Age-Associated Microbial Dysbiosis Promotes Intestinal Permeability, Systemic Inflammation, and Macrophage Dysfunction. Cell Host Microbe 2017;21:455-466 e4.
9. Burns RC, Rivera-Nieves J, Moskaluk CA, et al. Antibody blockade of ICAM-1 and VCAM-1 ameliorates inflammation in the SAMP-1/Yit adoptive transfer model of Crohn's disease in mice. Gastroenterology 2001;121:1428-36.
10. Kodani T, Rodriguez-Palacios A, Corridoni D, et al. Flexible Colonoscopy in Mice to Evaluate the Severity of Colitis and Colorectal Tumors Using a Validated Endoscopic Scoring System. Jove-Journal of Visualized Experiments 2013.
11. Corridoni D, Kodani T, Rodriguez-Palacios A, et al. Dysregulated NOD2 predisposes SAMP1/YitFc mice to chronic intestinal inflammation. Proc Natl Acad Sci U S A 2013;110:16999-7004.
12. Rodriguez-Palacios A, Aladyshkina N, Ezeji JC, et al. 'Cyclical Bias' in Microbiome Research Revealed by A Portable Germ-Free Housing System Using Nested Isolation. Scientific Reports 2018;8:18.
13. Connors J, Dawe N, Van Limbergen J. The Role of Succinate in the Regulation of Intestinal Inflammation. Nutrients 2018;11.
14. Morotomi M, Nagai F, Sakon H, et al. Paraprevotella clara gen. nov., sp. nov. and Paraprevotella xylaniphila sp. nov., members of the family 'Prevotellaceae' isolated from human faeces. Int J Syst Evol Microbiol 2009;59:1895-900.
15. Nickerson KP, Chanin R, McDonald C. Deregulation of intestinal anti-microbial defense by the dietary additive, maltodextrin. Gut Microbes 2015;6:78-83.

16. Pandit M, Acharya S, Gu Y, et al. Geranylgeranyl pyrophosphate amplifies T(reg) differentiation via increased IL-2 expression to ameliorate DSS-induced colitis. Eur J Immunol 2021;51:1461-1472.
17. Wang Z, Tian L, Jiang Y, et al. Synergistic role of gut-microbial L-ornithine in enhancing ustekinumab efficacy for Crohn's disease. Cell Metab 2025;37:1089-1102 e7.
18. Ghiboub M, Penny S, Verburgt CM, et al. Metabolome Changes With Diet-Induced Remission in Pediatric Crohn's Disease. Gastroenterology 2022;163:922-936 e15.
19. Chiaro TR, Soto R, Zac Stephens W, et al. A member of the gut mycobiota modulates host purine metabolism exacerbating colitis in mice. Sci Transl Med 2017;9.
20. Crittenden S, Cheyne A, Adams A, et al. Purine metabolism controls innate lymphoid cell function and protects against intestinal injury. Immunol Cell Biol 2018;96:1049-1059.
21. Johansson JE, Soussi B, Bagge U, et al. Disturbance of purine nucleotide metabolism: a possible early key event in development of intestinal damage induced by chemotherapy. Dig Dis Sci 2001;46:257-61.
22. Bersch KL, DeMeester KE, Zagani R, et al. Bacterial Peptidoglycan Fragments Differentially Regulate Innate Immune Signaling. ACS Cent Sci 2021;7:688-696.
23. Akiyama S, Nishijima S, Kojima Y, et al. Multi-biome analysis identifies distinct gut microbial signatures and their crosstalk in ulcerative colitis and Crohn's disease. Nat Commun 2024;15:10291.
24. Ananthakrishnan AN, Luo C, Yajnik V, et al. Gut Microbiome Function Predicts Response to Anti-integrin Biologic Therapy in Inflammatory Bowel Diseases. Cell Host Microbe 2017;21:603-610 e3.
